# Mortality Statistics

**Published:** 1874-10

**Authors:** 


### *Chicago Mortality Report for August, 1874.*

MORTALITY IN MONTH OF AUGUST, 1874.

| | |
|---|---|
| Accident, by burns | 4 |
| " by being crushed | 2 |
| " by fall | 5 |
| " by drowning | 13 |
| " by lightning | 1 |
| " run over by wagon | 1 |
| " by suffocation | 1 |
| " by shooting | 1 |
| " by railroad | 1 |
| Abscess, hip | 1 |
| " lumbar | 1 |
| " cerebral | 1 |
| Anæmia | 1 |
| Apoplexy | 6 |
| Arachnitis | 3 |
| Asthma | 2 |
| Atelectasis pulmonum | 3 |
| Bowels, congestion of | 2 |
| " obstruction of | 1 |
| Brain, congestion of | 7 |
| " inflammation of | 14 |
| " softening of | 3 |
| Bronchitis | 3 |
| " capillary | 1 |
| Cancer of breast | 1 |
| " of bowels | 1 |
| " of liver | 1 |
| " of stomach | 5 |
| " of uterus | 2 |
| Cholera infantum | 406 |
| " morbus | 7 |
| Consumption | 43 |
| Convulsions | 126 |
| " puerperal | 2 |
| Cyanosis | 1 |
| Cystitis | 1 |
| Cynanche trachealis | 1 |
| Debility, general | 13 |
| Delirium tremens | 1 |
| Diarrhœa | 77 |
| " chronic | 8 |
| Diphtheria | 10 |
| Dropsy, general | 8 |
| Dysentery | 19 |
| Dysentery, typhoid | 1 |
| Empyema | 1 |
| Enteritis | 37 |
| Entero colitis | 27 |
| Epilepsy | 3 |
| Exhaustion | 2 |
| Erysipelas | 3 |
| Fever, congestive | 1 |
| " intermittent | 1 |
| " puerperal | 3 |
| " remittent | 4 |
| " scarlet | 11 |
| " typhoid | 28 |
| Gastritis | 5 |
| Gastro enteritis | 15 |
| Heart, disease of | 7 |
| " fatty degeneration of | 3 |
| " rupture of | 1 |
| " enlargement of | 1 |
| " valvular disease of | 7 |
| Hemiplegia | 1 |
| Hepatitis | 3 |
| Hernia | 2 |
| Hydrophobia | 1 |
| Hydrocephalus | 16 |
| Hydrothorax | 1 |
| Inanition | 39 |
| Illeo enteritis | 1 |
| " colitis | 1 |
| Intemperance | 2 |
| Intussusception | 1 |
| Influenza | 1 |
| Injury at birth | 1 |
| Kidneys, Bright's disease of | 3 |
| Liver, disease of | 3 |
| " cirrhosis of | 1 |
| " fatty degeneration of | 1 |
| " hypertrophy of | 1 |
| Lungs, congestion of | 3 |
| " hemorrhage of | 1 |
| " paralysis of | 1 |
| Leuchæmia | 1 |
| Malformation | 1 |
| Menstrual suppression | 1 |

| | | | |
|---|---|---|---|
| Mania, puerperal | 1 | Septicæmia | 1 |
| Measles | 2 | Small-Pox | 1 |
| Meningitis | 20 | Stomach, ulceration of | 1 |
| " cerebro-spinal | 13 | Stomatitis | 1 |
| " tubercular | 5 | Suicide, by drowning | 1 |
| Mouth, canker sore of | 1 | " by laudanum | 1 |
| Œdema pulmonum | 1 | " by hanging | 1 |
| Old age | 13 | Tabes mesenterica | 23 |
| Otitis | 1 | Teething | 9 |
| Paralysis | 2 | Teething and complications | 7 |
| Parotitis | 1 | Tetanus | 1 |
| Pericarditis | 1 | Trismus | 1 |
| Peritonitis | 4 | " nascentum | 1 |
| " puerperal | 1 | Tumor | 1 |
| Pneumonia | 11 | Uterus, hemorrhage of | 2 |
| Pleurisy | 2 | " degeneration of | 1 |
| Pyæmia | 1 | Vitality deficient | 1 |
| Rheumatism, inflammatory | 1 | Whooping cough | 21 |
| Sclerosis of spinal cord | 1 | Total | 1,220 |
| Premature births, 7; Still births, 58. | | Total | 65 |

COMPARISON.

| | | |
|---|---|---|
| Deaths in month of August, 1874 | | 1,220 |
| " " July, 1874 | | 1,454 |
| Decrease | 234 | |
| Deaths in month of August, 1873 | | 1,423 |
| Decrease | 203 | |

AGES.

| | | | |
|---|---|---|---|
| Under one year | 567 | Forty years to fifty | 41 |
| One year to two | 309 | Fifty " " sixty | 37 |
| Two years to three | 24 | Sixty " " seventy | 26 |
| Three " " four | 14 | Seventy " " eighty | 18 |
| Four " " five | 7 | Eighty " " ninety | 6 |
| Five " " ten | 19 | Ninety " " one hundred | -- |
| Ten " " twenty | 29 | | |
| Twenty " " thirty | 56 | Total | 1,220 |
| Thirty " " forty | 67 | | |

| | | | |
|---|---|---|---|
| Colored | 11 | Males | 670 |
| White | 1,209 | Females | 550 |
| Total | 1,220 | Total | 1,220 |

Married, 197; Single, 1023. Total 1,220

NATIVITIES, AUGUST, 1874.

| | | | |
|---|---|---|---|
| Belgium | 2 | Ireland | 58 |
| Bohemia | 4 | India | 1 |
| Canada | 7 | Italy | 2 |
| Native—Chicago | 154 | New Foundland | 1 |
| Foreign, " | 725 | Norway | 11 |
| United States, other parts | 107 | Nova Scotia | 1 |
| Denmark | 6 | Poland | 4 |
| England | 7 | Scotland | 3 |
| France | 1 | Sweden | 12 |
| Germany | 103 | Unknown | 10 |
| Holland | 1 | Total | 1,220 |

Deaths daily, 39⅓. Mean temperature, 72°. Rain fall, 3.15 inches.

MORTALITY BY WARDS, AUGUST, 1874.

| Wards. | No. Deaths. | Pop. in 1872. | | | Percentage. |
|---|---|---|---|---|---|
| 1 | 4 | 398 | one death in | | .... |
| 2 | 8 | 2,174 | " | " | .... |
| 3 | 23 | 19,157 | " | " | 833 |
| 4 | 21 | 16,832 | " | " | 801 |
| 5 | 39 | 18,564 | " | " | 476 |
| 6 | 125 | 31,371 | " | " | 251 |
| 7 | 143 | 27,644 | " | " | 193 |
| 8 | 120 | 30,253 | " | " | 252 |
| 9 | 77 | 30,032 | " | " | 390 |
| 10 | 24 | 16,624 | " | " | 693 |
| 11 | 33 | 18,340 | " | " | 556 |
| 12 | 42 | 20,876 | " | " | 497 |
| 13 | 36 | 14,636 | " | " | 406 |
| 14 | 42 | 15,892 | " | " | 378 |
| 15 | 170 | 40,047 | " | " | 235 |
| 16 | 69 | 19,099 | " | " | 278 |
| 17 | 71 | 17,513 | " | " | 246 |
| 18 | 66 | 19,977 | " | " | 302 |
| 19 | 7 | 2,944 | " | " | .... |
| 20 | 18 | 5,023 | " | " | .... |
| | 1,138 | Ratio of deaths to population in 1872, one death in 310. | | | |

| | | | |
|---|---|---|---|
| No. deaths in Wards | 1,138 | Home for Friendless | 1 |
| Accidents | 34 | Mercy Hospital | 3 |
| Bridewell | 1 | St. Joseph's " | 7 |
| County Hospital | 11 | St. Luke's " | 3 |
| Foundlings' Home | 12 | Suicides | 3 |
| Hospital Alexian Brothers | 5 | | |
| Hospital for Women and Child'n | 2 | Total | 1,220 |

